# MicroRNA 21 Is a Homeostatic Regulator of Macrophage Polarization and Prevents Prostaglandin E_2_-Mediated M2 Generation

**DOI:** 10.1371/journal.pone.0115855

**Published:** 2015-02-23

**Authors:** Zhuo Wang, Stephanie Brandt, Alexandra Medeiros, Soujuan Wang, Hao Wu, Alexander Dent, C. Henrique Serezani

**Affiliations:** 1 Department of Microbiology and Immunology, Indiana University School of Medicine, Indianapolis, Indiana, United States of America; 2 Departamento de Ciências Biológicas, Faculdade de Ciências Farmacêuticas, Universidade Estadual Paulista “Júlio de Mesquita Filho,” 14801–902 Araraquara, São Paulo, Brazil; Fundação Oswaldo Cruz, BRAZIL

## Abstract

Macrophages dictate both initiation and resolution of inflammation. During acute inflammation classically activated macrophages (M1) predominate, and during the resolution phase alternative macrophages (M2) are dominant. The molecular mechanisms involved in macrophage polarization are understudied. MicroRNAs are differentially expressed in M1 and M2 macrophages that influence macrophage polarization. We identified a role of miR-21 in macrophage polarization, and found that cross-talk between miR-21 and the lipid mediator prostaglandin E_2_ (PGE_2_) is a determining factor in macrophage polarization. miR-21 inhibition impairs expression of M2 signature genes but not M1 genes. PGE_2_ and its downstream effectors PKA and Epac inhibit miR-21 expression and enhance expression of M2 genes, and this effect is more pronounced in miR-21-/- cells. Among potential targets involved in macrophage polarization, we found that STAT3 and SOCS1 were enhanced in miR-21-/- cells and further enhanced by PGE_2_. We found that STAT3 was a direct target of miR-21 in macrophages. Silencing the STAT3 gene abolished PGE_2_-mediated expression of M2 genes in miR-21-/- macrophages. These data shed light on the molecular brakes involved in homeostatic macrophage polarization and suggest new therapeutic strategies to prevent inflammatory responses.

## Introduction

Macrophages are pleiotropic cells that can function as immune effectors and regulators, tissue remodelers, or scavengers [1]. The acute inflammatory response is characterized by the presence of M1 macrophages, and the chronic or resolution inflammatory phases are mediated by the enrichment of M2 macrophages [2]. While microbial products such as LPS and the proinflammatory cytokines TNF-α and IFN-induce M1 macrophages, M2 macrophages are generated in the presence of IL-4, IL-13 or IL-10 [3]. M1 are known to enhance microbial clearance and to enhance cell recruitment to the inflammatory focus by secreting TNF-α, IL-1β, and nitric oxide, and M2 macrophages are known to enhance fungal phagocytosis and to secrete pro-resolution substances including fibronectin, IL-10, TGF-β, and metalloproteases [2].

Prostaglandin E_2_ (PGE_2_) is an endogenous lipid mediator produced in abundance at sites of inflammation and infection, and itself modulates many aspects of macrophage function [4–6]. The immunomodulatory effects of PGE_2_ in macrophages largely result from its ability to increase intracellular cAMP through the stimulatory G protein (G_s_)-coupled E prostanoid (EP) receptors EP2 and EP4 [7]. Increases in intracellular cAMP levels activate two downstream effector molecules, cAMP-dependent protein kinase A (PKA) and the exchange protein directly activated by cAMP-1 (Epac-1) [7–10]. Increases in intracellular cAMP generally suppress innate immune functions of macrophages, including suppressing the generation of inflammatory mediators such as TNF-α and the phagocytosis and killing of microbes [4,7,8,11–14]. However, the role of PGE_2_ in the generation of M1 or M2 macrophages is poorly understood. Depending on the cell type investigated, the PGE_2_/EP2-EP4/cAMP/PKA cascade has been shown to enhance, inhibit, or exert no effect on iNOS expression [9], an M1 marker, and PGE_2_ has been shown to enhance the production of IL-10 and IL-6 [9], which generates M2 macrophages. The molecular mechanisms by which PGE_2_ enhances the generation of M2 macrophages remain to be determined.

MicroRNAs are small oligonucleotides (∼ 22 nt) that bind complementary sequences in mRNAs, usually resulting in gene silencing via translational repression or target degradation [15]. Recent studies have shown that microRNAs are key determinants of macrophage activation, controlling the expression of a variety of molecules involved in PRR signaling and NFκB activation [15–23]. Different microRNAs are expressed in M1 or M2 polarized macrophages and have been shown to control macrophage polarization [24–27, 28,29]. The role of miR-21 in macrophage polarization is unknown but numerous properties of miR21 have been described. miR-21 expression is enhanced by inflammatory stimuli, including LPS stimulation. miR-21 impairs MyD88-dependent NFκB activation and IL-6 expression, but enhances IL-10 expression [30]. miR-21 targets the proinflammatory molecule tumor suppressor programmed cell death protein 4 [30]. miR-21 targets other proinflammatory cytokines, such as IL-12p35, during allergic inflammation [31]. miR-21 -/- mice exhibit lower levels of TNF-α, MIP-2, and NFκB p65 in a model of fatal colitis [32].

Since PGE_2_ exerts anti-inflammatory effects in macrophages by inhibiting TNF-α and enhancing IL-10 levels [14,33], and lack of miR-21 exhibits similar effects, we sought to investigate possible interplay between miR-21 and PGE_2_ that might lead to macrophage polarization. We found that macrophages from miR-21 deficient mice are enriched in M2 macrophages and depleted of M1 macrophages. Furthermore, miR-21 deficiency potentiates PGE_2_-mediated M2 macrophages in a STAT3-dependent manner.

## Results

### miR-21 drives M1 and inhibits M2 peritoneal macrophage polarization

**Fig 1:**
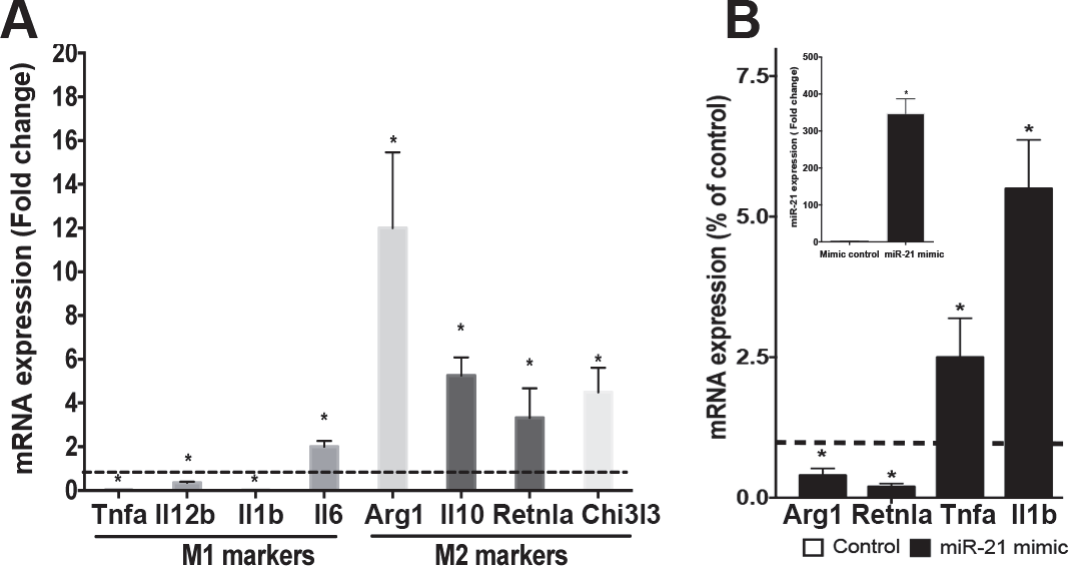
miR-21 deficiency impairs M1 and favors M2 macrophage polarization. **(A)** Thioglycollate-elicited macrophages from WT and miR-21 deficient mice were isolated, and the expression of *Tnfa, Il12p40, Il1b, Arg1, Il10, IL-6, Retnla, Chi3l3*, and *Actin* mRNA was determined by real time PCR. **(B)** Elicited macrophages were transfected with the miR-21mimic or the scrambled control (30 nM each), and the expression of *Retnla, Arg1*,*Tnfa, Il1b* and *Actin* was determined by real time PCR. Inset: miR-21 mRNA expression of macrophages transfected as in **B**. Data represent mean ± SEM from 3–5 individual experiments, each performed in triplicate. *p < 0.05 versus WT cells or mimic control.

The role of miR-21 in the control of macrophage polarization remains to be determined. Initially we determined the expression profile of M1 and M2 markers by real time PCR in thioglycollate-elicited macrophages. Our data showed that the M1 markers *Tnfa, Il12p40, Il1b*, and *Il6* were either not detected or were detected at lower levels than observed in WT macrophages **(Fig. 1A)**. We found that all M2 markers studied (*Il10, Arg1, Retnla*, and *Chi3l3*) were enhanced in miR-21-/- macrophages when compared to WT macrophages **(Fig. 1A)**. These same genotype-specific patterns of expression were observed in resident peritoneal and alveolar macrophages (data not shown). Thus, further experiments were performed only in elicited macrophages. We confirmed that miR-21 inhibited the expression of genes present in M2 macrophages and enhances the expression of genes enriched in M1 macrophages by treating WT cells with the miR-21 mimic **(Fig. 1B and inset)**. Our findings indicate that miR-21 is a homeostatic regulator of macrophage polarization even in the absence of conventional M1 or M2 stimuli.

### PGE_2_ inhibits miR-21 expression in macrophages

**Fig 2:**
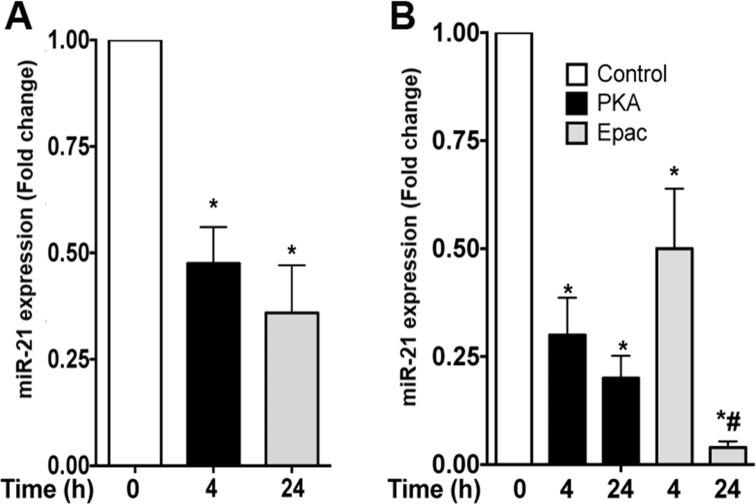
PGE_2_/PKA/Epac axis inhibits miR-21 expression in macrophages. Elicited macrophages were treated or not with 1 μM PGE_2_ **(A)** or 500 μM of PKA-specific cAMP analog 6-Bnz-cAMP or Epac-specific cAMP analog 8-pCPT-2-O-Me-cAMP each **(B)** for the indicated times, and the expression of miR-21 was determined by real time PCR. Data represent mean ± SEM from 3–5 individual experiments, each performed in triplicate. *p < 0.05 versus unstimulated cells; # p<0.05 versus cells stimulated with PKA.

Previously, we have shown that PGE_2_ enhances *Il10* and *Il6* production in a PKA dependent manner [14,34]. However, the role of PGE_2_ and its downstream effectors in macrophage polarization is poorly understood. Furthermore, whether PGE_2_ controls microRNA expression remains to be determined. We found that PGE_2_ inhibited miR-21 expression both at 4 and 24 h of stimulation compared to untreated cells **(Fig. 2A)**. Next, we sought to determine which cAMP downstream effector, PKA or Epac, inhibited miR-21 expression. We stimulated macrophages with the PKA agonist 6-Bnz-cAMP or Epac agonist 8-pCPT-2-*O*-Me-cAMP for different times and found that both effectors inhibited miR-21 expression. However, while PKA activation was more potent in inhibiting miR-21 at earlier time points, Epac activation was more effective in reducing miR-21 expression after 24 h of activation **(Fig. 2B)**. These results suggest that distinct cAMP effectors that influence different transcriptional programs differentially inhibit miR-21 expression.

### miR-21 is an endogenous brake in PGE_2_-mediated M2 polarization

**Fig 3:**
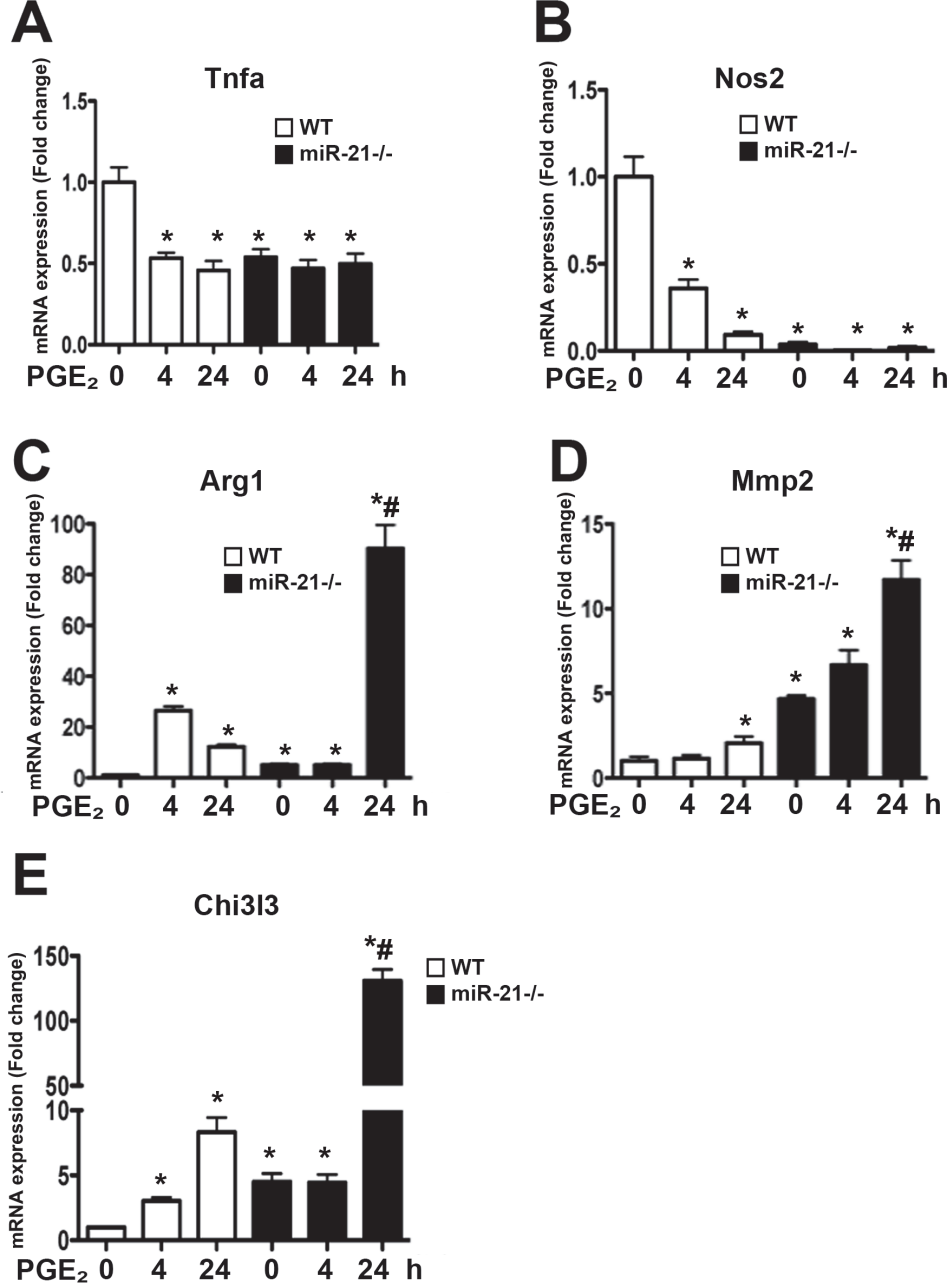
Absence of miR-21 amplifies PGE_2_-mediated M2 polarization. Thioglycollate-elicited macrophages from WT and miR-21 deficient mice were stimulated or not with PGE_2_ for 4 or 24 h, and the expression of *Tnfa, Nos2, Arg1, Mmp2*, and *Chi3l3* mRNA was determined by real time PCR. Data represent mean ± SEM from 3–5 individual experiments, each performed in triplicate. *p < 0.05 versus unstimulated cells and p<0.05 versus WT macrophages; # p < 0.05 versus WT cells stimulated with PGE_2_ for 24h.

To study the profile of macrophage polarization in PGE_2_-stimulated macrophages, WT cells were challenged with PGE_2_ for 4 and 24 h, and the profiles of M1 and M2-related genes in macrophages were determined by real time PCR. We found that PGE_2_ decreased the expression of M1 markers *Tnfa* and *Nos2* **(Fig. 3 A and B)** and enhanced the expression of M2 markers Arg1, *Mmp2*, and *Chi3l3*. While Arg1 expression was more pronounced 4 h after PGE_2_ stimulation, the expression of *Mmp2* and *Chi3l3* were higher 24 h after PGE_2_ stimulation **(Fig. 3 C-E)**. When miR-21 deficient macrophages were tested, we observed that PGE_2_ further enhanced M2 markers 24h after stimulation compared to M2 marker expression in WT cells **(Fig. 3 C-E)**. These data suggest that PGE_2_ enhances M2 markers, and that miR-21 acts as an endogenous brake involved in the PGE_2_-mediated generation of M2 cells.

### miR-21 targets STAT3 to inhibit M2 polarization

**Fig 4:**
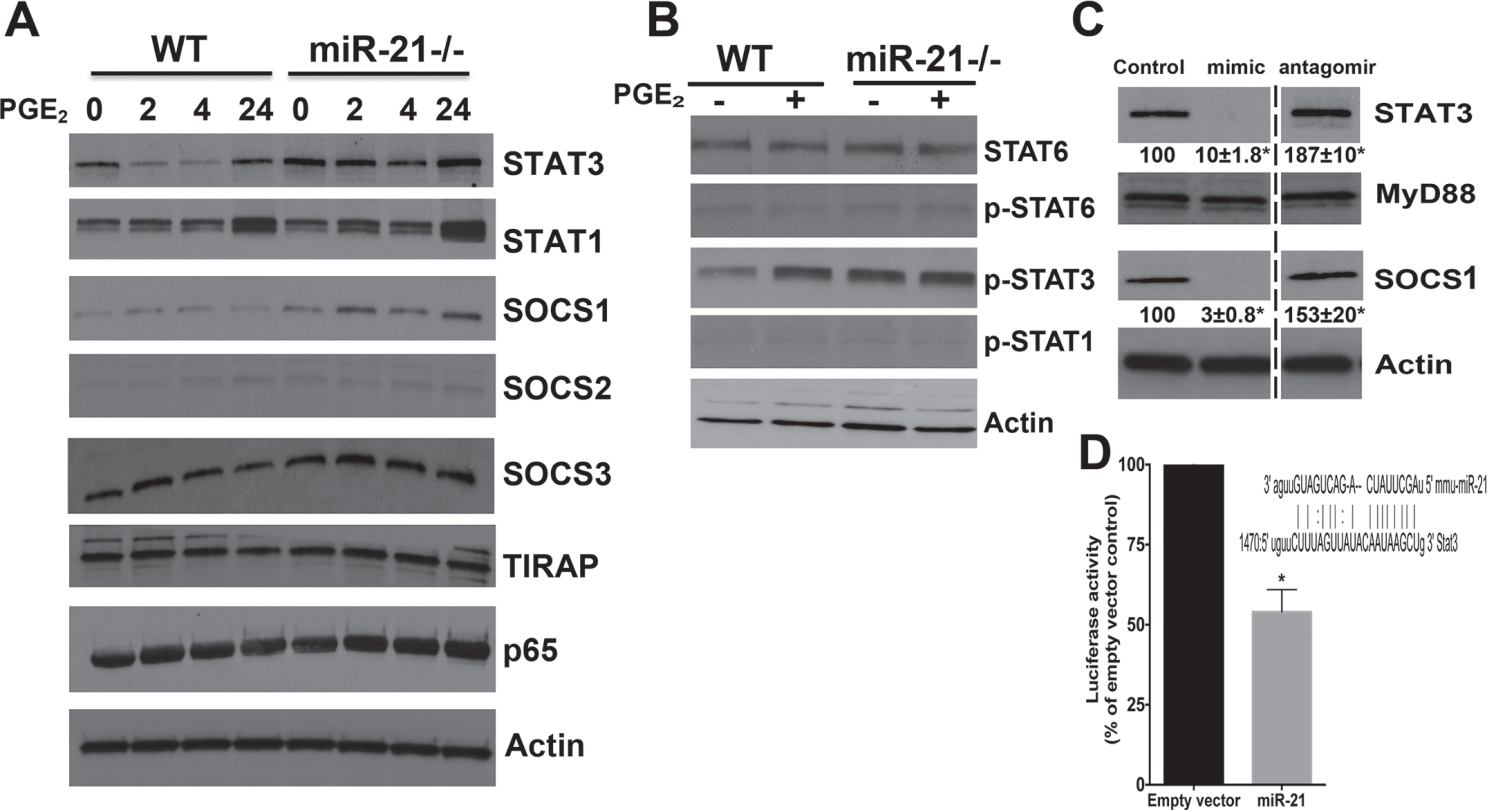
miR-21 targets STAT3 in macrophages. **(A)** Thioglycollate-elicited macrophages from WT and miR-21 deficient mice were stimulated or not with PGE_2_ for the indicated times, and the expression of STAT3, STAT1, SOCS1, SOCS2, SOCS3, TIRAP, NFκB p65, and beta actin was determined by immunoblotting. **(B)** Elicited macrophages from WT and miR-21 deficient mice were stimulated or not with PGE_2_ for 24 h, and the expression of t-STAT6, pSTAT6 (Tyr641), pSTAT1 (Tyr701), pSTAT3 (Tyr705) and beta actin was determined by immunoblotting. **(C)** Elicited macrophages from WT were transfected with 30 nM of scrambled control, miR-21, or miR-21 antagomir for 24 h, followed by determination of STAT3, MyD88, SOCS1, and beta-actin by immunoblotting. The numbers represent mean densitometric analysis of the bands shown from 3 independent experiments. The dashed line in the figure indicates lanes in the membrane that contained experimental conditions that were run under the same experimental conditions but not pertinent and were omitted. Data are mean ± SEM; *p < 0.01 versus WT scrambled control-treated cells. **(D)** Raw264.7 macrophages were transfected with a luciferase construct containing the 3′ UTR of STAT3 and empty vector expressing luciferase reporter plasmid followed by the microRNA mimic miR-21 (30 nM) for 24 h, and luciferase activity was determined. Inset: The predicted miR-21 seed sequence located in the 3′-UTR of STAT3. Sequence alignment of miR-21 and STAT3 is shown, and matches are indicated by a line. Data represent mean ± SEM from at least 3 individual experiments, each performed in triplicate. *p < 0.05 versus empty vector.

To understand the mechanisms involved in miR-21 inhibition of macrophage polarization, we initially determined the expression profiles of proteins and transcription factors involved in M1 and M2 polarization. We found that 24 h after PGE_2_ challenge the expression of STAT3 and STAT1 but not STAT6 was enhanced in WT macrophages **(Fig. 4 A and B)**. We also observed an increase in STAT3 expression in miR-21 -/- macrophages, which PGE_2_ treatment enhanced further **(Fig. 4 A)**. We did not observe changes in the expression of NFκB p65, SOCS3, MyD88, or TIRAP **(Fig. 4 A)**. We found that PGE_2_ treatment enhanced SOCS-1 expression levels in both miR-21 -/- and WT macrophages **(Fig. 4 A)**. Enhanced STAT3 expression also correlated with increased STAT3 phosphorylation, but not STAT1 and STAT6 activation.

To further confirm that miR-21 targeted STAT3, we determined the expression of STAT3 in WT macrophages treated with miR-21 mimic or with the miR-21 antagomir **(Fig. 4C)**. The miR-21 mimic inhibited STAT3 expression, and the antagomir further enhanced STAT3 expression **(Fig. 4 C)**, indicating the miR-21 targeted STAT3 expression. We then look at the protein expression of the M2 marker SOCS1 and the M1 marker SOCS3 [35–37]. We found that the miR-21 mimic inhibited SOCS-1 expression while the antagomir enhanced SOCS-1 expression. Neither the mimic nor antagomir affected the expression of MyD88 **(Fig. 4 C)**. To further confirm that miR-21 directly targeted STAT3 3’UTR sequences, we transfected RAW 264.6 cells with a plasmid expressing STAT3–3’UTR luciferase or empty vector, followed by transfection of cells with miR-21 mimic or scrambled mimic. We found that miR-21 directly inhibited STAT3 expression, indicating that miR-21 targeted STAT3–3’UTR sequences **(Fig. 4 D)**. The inset shows the predicted miR-21 seed sequence in the STAT3–3’UTR **(www.microRNA.org; Fig. 4 D)**.

### STAT3 mediates PGE_2_-induction of M2 macrophages in miR-21 deficient macrophages

**Fig 5:**
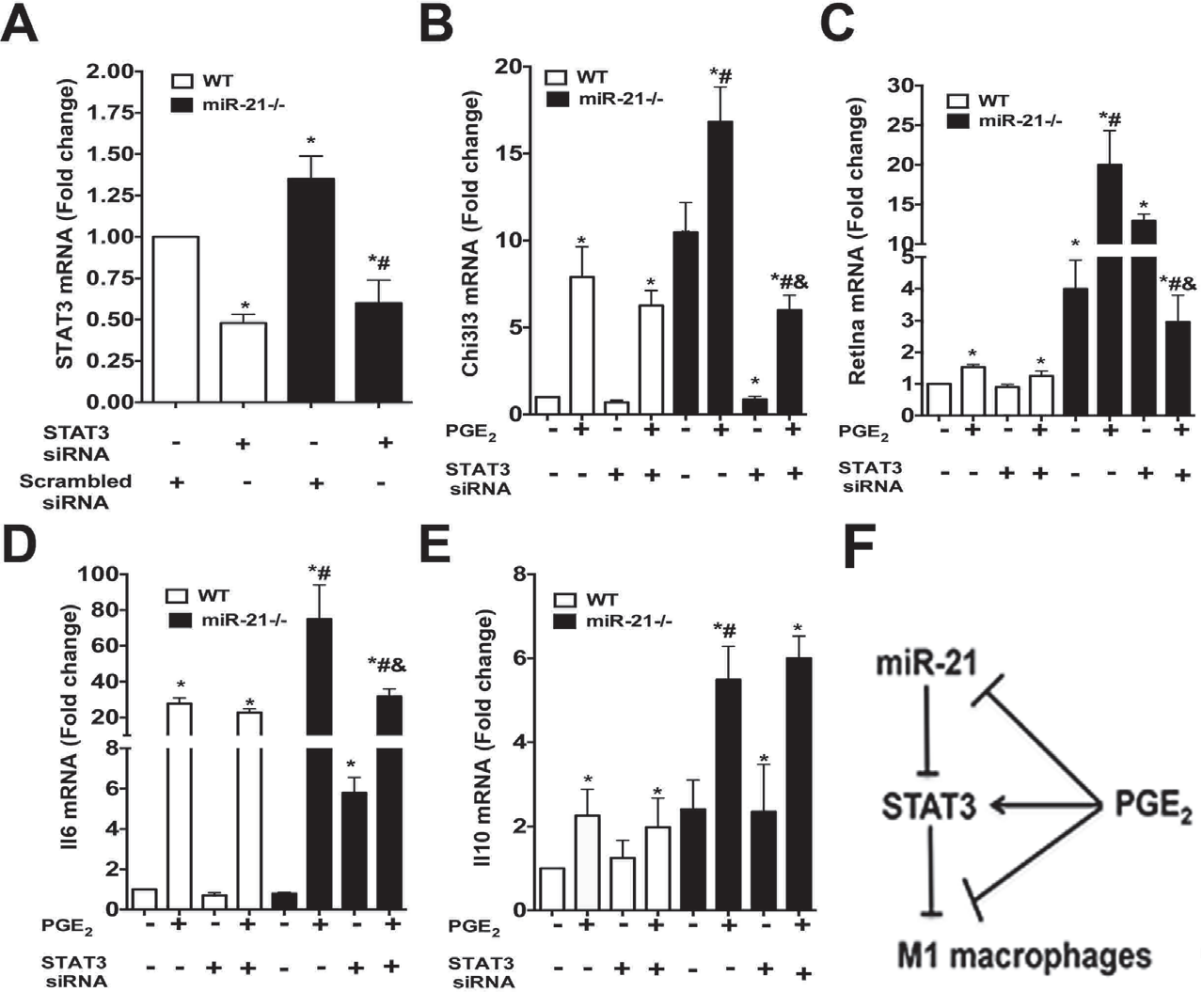
PGE_2_ utilizes STAT3 signaling to enhance M2 macrophages in miR-21 deficient macrophages. WT and miR-21 deficient macrophages were treated with 30 nM STAT3 siRNA or siRNA control for 24 h and stimulated or not with PGE_2_ for another 24 h. Expression of **(A)** *Stat3*, **(B)** *Chi3l3*, **(C)** *Retnla*, and **(D)** *Il6*, **(E)** *Il10* mRNA were determined by real time PCR. **(F)** Proposed model of miR-21 and PGE2-mediated M2 macrophage generation. Data represent mean ± SEM from at least 3 individual experiments, each performed in triplicate. *p < 0.05 versus WT siControl; ^#^p<0.05 versus miR-21 -/- cells and ^&^p<0.05 versus STAT3 siRNA control.

Since STAT3 expression was enhanced in miR-21 -/- macrophages and PGE_2_ stimulation further enhanced STAT3 in these cells, we determined whether enhanced STAT3 expression in miR-21 -/- cells stimulated with PGE_2_ influenced M2 polarization. We silenced STAT3 in both WT and miR-21 deficient cells (Fig. 5A). We then stimulated with PGE_2_ and determined expression of M2-markers by real time PCR **(Fig. 5B-E)**. When STAT3 was silenced in WT macrophages, we did not observe changes in M2 markers **(Fig. 5B-E)**. Furthermore, STAT3 depletion did not prevent expression of M2 genes in PGE_2_-stimulated WT macrophages, indicating that STAT3 does not mediate homeostatic M2 polarization **(Fig. 5B-E)**. While Stat3 silencing did not change the expression of M2 markers in WT cells, Stat3 inhibition in miR-21 -/- macrophages inhibited the expression of *Chi3l3* and enhanced *Retnla* and *Il6* mRNA expression. While Stat3 silencing abolished PGE_2_-enhanced expression of *Chi3l3, Retnla*, and *Il6* in miR-21 -/- cells, we did not observe an effect of *Stat3* inhibition on *Il10* expression. These results indicate that increased M2 polarization in PGE_2_-stimulated miR-21 -/- cells is dependent on STAT3 signaling.

## Discussion

This study highlights a novel regulatory mechanism of homeostatic M2 macrophage polarization along the PGE_2_/cAMP axis mediated by miR-21 **(Fig. 5F)**. We and others have shown that PGE_2_ inhibits TNF-α production and enhances IL-10 levels in macrophages from different anatomical sites [4,7,14,38]. miR-21 deficiency also leads to low TNF-α and enhanced IL-10 production in macrophages [30,39]. Here, we extended these findings by investigating cross-talk between PGE_2_ and miR-21 in the development of macrophage anti-inflammatory effects and the molecular programs involved. We found that: 1) miR-21 deficiency inhibits the expression of M1 and enhanced M2 markers in elicited peritoneal macrophages; 2) PGE_2_/PKA/Epac inhibited miR-21 expression; 3) PGE_2_ enhances the expression of M2 markers, and its effects are enhanced in miR-21 deficient cells; 4) miR-21/PGE_2_ targets STAT3 and SOCS1, but not STAT1, STAT6, SOCS3, or TLR adaptors; 5) STAT3 silencing prevents PGE_2_-induced M2 marker expression in miR-21 deficient cells.

M1 macrophages are classically induced by IFN-γ and LPS, and M2 macrophages are induced by IL4 and IL-13 [39]. Furthermore, M2 macrophages can be further classified in M2a, M2b and M2c, depending on the stimuli. Here, we show that depletion of a single microRNA led to inhibition of M1 and enhanced M2 markers in the absence of any stimuli, indicating that miR-21 functions to keep macrophage polarization in check, and depletion of this microRNA favors the expression of M2 markers. Whether miR-21 differentially regulates specific M2 populations remains to be determined. The results showed that effects of miR-21 in the deficient mice were not the consequence of a putative compensatory mechanism that might influence the expression of different microRNAs. That miR-21 controls LPS actions has been shown. Sheedy et al have shown that miR-21 inhibits the production of IL-6 and enhances IL-10 levels by controlling both NFκB p65 levels and the proinflammatory molecule tumor suppressor programmed cell death 4 (PDCD4), an inhibitor of IL-10 production [15,30]. These findings are not in perfect agreement with ours, since we found that miR-21 -/- elicited macrophages show enhanced IL-6 and IL-10 levels, whereas Sheedy et al. show that miR-21 mimic enhances IL-6 and decrease IL-10 levels [30]. The reason for this discrepancy is uncertain, but there are several differences in the respective experiments. Sheedy et al studied miR-21 effects in a macrophage cell line and bone marrow derived macrophages; whereas, we performed our experiments in thioglycollate-elicited macrophages, which exhibit an inflammatory phenotype [30]. Also, we investigated the role of miR-21 in basal/homeostatic expression of these cytokines, and Sheedy et al determined the production of IL-16 and IL-10 in LPS-stimulated cells [30]. Whether thioglycollate-elicited miR-21 deficient macrophages respond to LPS in the same manner as cell lines or bone marrow macrophages remains to be determined. Our results are in agreement with Shi et al. who showed in a model of colitis that M1-like cytokines such as TNF-αand MIP-2 are decreased in miR-21 deficient mice [32]. Clearly, the elucidation of such cell-specific functions of miR-21 should help in the development of effective miR-based therapeutic strategies.

Here, we investigated the role of the cAMP inducer PGE_2_ in the expression of miR-21 in macrophages. Our data show that PGE_2_ challenge decreased miR-21 expression, and incubation of macrophages with the downstream effectors PKA or the Epac agonist also decreased miR-21 levels. Whether the cAMP/PKA/Epac axis influences global microRNA expression and their effects in macrophage biology remains to be determined. These data lead us to speculate that miR-21 acts as a brake on PGE_2_ effects, and PGE_2_-mediated miR-21 inhibition is part of an inhibitory loop involved in macrophage M2 polarization by the PGE_2_/PKA/Epac axis.

The molecular mechanisms involved in miR-21 and PGE_2_-induced M2 macrophages were studied. Initially, we tested whether miR-21 inhibition influenced EP 1–4 mRNA expression, but we did not observe any differences in the expression of these receptors between WT and miR-21 -/- cells or WT cells treated with miR-21 mimic (data not shown). We also did not observe any change in the expression of the CREB (data not shown). We next investigated which transcription factors and effectors were involved in enhanced expression of M2 genes. While STAT6 is activated by IL-4 and IL-13 [40], and has been suggested to be the master regulator of M2 differentiation, STAT1 and NFκB are thought to be essential for M1 generation [40]. We did not observe a change in the expression of STAT6 and phosphorylation in miR-21-/- macrophages, which led us to study the expression of other STAT proteins in miR-21 deficient cells, and to ask whether PGE_2_ further influenced the expression of these proteins. We found that STAT3 and STAT1 levels were enhanced in miR-21-/- macrophages, and treatment with PGE_2_ further enhanced STAT3 expression and phosphorylation, but not STAT1 activation when compared to WT cells. That miR-21 expression is controlled by STAT3 has been shown [41–43]; however, whether miR-21 controls STAT3 levels is unknown. We found that miR-21 -/- and miR-21 antagomir transfection enhanced STAT3 levels, and conversely, miR-21 mimic decreased STAT3. Whether miR-21 directly targets SOCS1 and whether STAT3 controls SOCS1 levels in a PGE_2_ dependent manner is under investigation.

PGE_2_ is known to control STAT3 activation and expression [44–47]. However, whether PGE_2_ utilizes STAT3 to inhibit macrophage function is poorly understood. Here, we found that Stat3 silencing abolished PGE_2_-enhanced expression of *Chi3l3, Retnla*, and *Il6* in miR-21 -/- cells, we did not observe an effect of Stat3 inhibition on *Il10* expression. The mechanisms involved in PGE_2_-induced STAT3 activation in macrophages remain to be determined. Frias et al, have shown that PGE_2_ enhances STAT3 phosphorylation in a manner dependent on ERK 1/2 activation but not on p38 MAPK activation [44,48]. Singh et al showed that PGE_2_ enhances STAT3 expression [49]. Another potential mechanism by which PGE_2_ enhances M2 dependent genes in a STAT3 dependent manner is through the production of Il6, which is known to induce STAT3 activation [50–52] and M2 generation [53]. Pretreatment of both PGE_2_ stimulated WT and miR-21 cells with anti-IL-6 did not prevent PGE_2_ effects on the expression of M2 genes (data not shown). Therefore, it still remains to be determined how PGE_2_ influences the expression of M2 dependent genes, and how miR-21 interferes with PGE_2_ signaling.

Effects of microRNAs on cytokine signaling and their consequences for macrophage biology may be a root cause of inflammation [24,54,55]. There are numerous approved pharmaceuticals that target STAT3. As well, specific microRNA mimics or antagomirs are also known to control the inflammatory response [24,54,55], and the use of such microRNAs to treat inflammatory diseases represents a potent therapeutic approach. In conditions were acute inflammation needs to be controlled, miR-21 antagomirs may be a good therapeutic agents that increase the numbers of M2 macrophages and decrease the expression of master inflammatory mediators such as IL-1β and TNF-α. Alternatively, miR-21 mimics could block chronic inflammatory responses by preventing M2 formation. In view of its role in inflammation, additional studies investigating the role of this microRNA in acute and chronic models of inflammation are warranted.

## Methods

### Animals

8-week-old female WT C57BL/6 mice (The Jackson Laboratory) or miR-21 deficient mice (miR-21 -/-) and counterpart WT [56] were maintained according to NIH guidelines for the use of experimental animals with the approval of the Indiana University School of Medicine Animal Care and Use Committees (Protocol Number: 10238). Surgical procedures were performed under isofluorane anesthesia and all necessary steps to minimize suffering were taken.

### Cell harvest and stimulation

Elicited macrophages were harvested from the peritoneal cavities of mice by lavage with PBS 4 days after the injection of 2 ml 3% thioglycollate as described previously [8]. Macrophages were stimulated with 1 μM PGE_2_ [14,33] or 500 μM PKA-specific cAMP analog 6-Bnz-cAMP (*N* ^6^-benzoyladenosine-3′,5′-cyclic monophosphate), or Epac-specific cAMP analog 8-pCPT-2-O-Me-cAMP (8–4-chlorophenylthio)-2′-O-methyladenosine-3′,5′-cyclic monophosphate) [12,14,34] for 24 h followed by RNA or protein isolation.

### Immunoblotting

Western blots were performed as previously described [8,9]. Protein samples were resolved by SDS-PAGE, transferred to a nitrocellulose membrane, and probed with commercially available primary antibodies against STAT3, STAT1, TIRAP, MyD88, NFκB p65, SOCS-1-3 (all at 1:500; Abcam), phosphorylated STAT3 (Tyr705), STAT1 (Tyr701) and STAT6 (Tyr641) (all at 1:1000; Cell Signaling) or **β**-actin (1:10,000; Sigma-Aldrich). Densitometric analysis was performed as described previously [8,9].

### RNA analysis

Total RNA from cultured cells was isolated using the miRNeasy Mini Kit (Qiagen) according to the manufacturer’s instructions. Quantitative RT-PCR analyses for miR-21 and RNU6 (used as a normalization control) were performed using TaqMan miRNA assays with reagents, primers, and probes obtained from Qiagen. In brief, a stem loop primer was used for reverse transcription (30 min, 16°C; 30 min, 42°C; 5 min 85°C) followed by qPCR employing TaqMan probes and primers in a Bio-rad CFX96 Mastercycler. For assessing expression of *Socs1, Myd88, Stat3, Il10, Chi3l3, Il6, Retnla, Nos2, Tnfa, Arg1, Mmp2*, and *actin*, cDNA was synthesized using a reverse transcription system (miScript II—Qiagem). qPCR was performed on a CFX96 Real-Time PCR Detection System (Bio-Rad Laboratories) as described [9]. Primers were purchased from Integrated DNA technologies. Relative expression was calculated using the comparative threshold cycle (Ct) and expressed relative to control or WT (ΔΔCt method).

### Targeted miRNA inhibition and overexpression

For inhibition of miR-21, macrophages were transfected using lipofectamine siRNA max transfection reagents with appropriate target anti-miR-21 antagomir or anti-miR-negative control 1 (anti-miR-control) [57]. For overexpression, macrophages were transfected with pre-miR-21 or pre-miR-negative control 1 (pre-miR-control) [57]. Transfection reagents, antagomirs, and control miRNAs were purchased from Invitrogen. Knockdown efficiency and overexpression efficiency were assessed by determining mature miR-21 levels in transfected cells. Transfected cells were treated with or without 30 nM microRNA mimic or antagomir for 48 h before collecting supernatants or preparing cell lysates for isolation of RNA (RNeasy kit, Qiagen) or proteins.

### Luciferase assays

Plasmids containing the 3′UTR of murine STAT3 were purchased from Genecopeia. The Raw 264.7 macrophage cell line was transfected with constructs in 6-well plates using lipofectamine siRNA max (Invitrogen). Firefly luciferase reporter gene constructs (0.1 μg per well) were co-transfected together with 30 nM of miR-21 or scrambled-miR control; cells were lysed 24 h after transfection, and luciferase activity was measured. Each sample was assayed in triplicate as described [57].

### Statistics

Data are presented as mean ± SEM. Comparisons among groups were assessed with ANOVA followed by Bonferroni analysis; *p* < 0.05 was considered significant.
